# Postpartum Hemorrhage-Induced Acute Kidney Injury Following Obstetric Hysterectomy: A Case Report

**DOI:** 10.7759/cureus.80516

**Published:** 2025-03-13

**Authors:** Anna Thanasa, Efthymia Thanasa, Emmanouil M Xydias, Evangelos Kamaretsos, Gerasimos Kontogeorgis, Ioannis Paraoulakis, Apostolos C Ziogas, Ioannis Thanasas

**Affiliations:** 1 Department of Health Sciences, Aristotle University of Thessaloniki, Thessaloniki, GRC; 2 Department of Obstetrics and Gynecology, EmbryoClinic IVF, Thessaloniki, GRC; 3 Department of Obstetrics and Gynecology, University General Hospital "Attikon" Medical School, National and Kapodistrian University of Athens, Trikala, GRC; 4 Department of Obstetrics and Gynecology, General Hospital of Trikala, Trikala, GRC; 5 Department of Obstetrics and Gynecology, University of Thessaly, Larissa, GRC

**Keywords:** acute kidney injury, case report, diagnostic findings, hemodialysis, obstetric hysterectomy, postpartum hemorrhage, pregnancy, prognosis

## Abstract

Pregnancy-related acute kidney injury (AKI) is rare, with hemorrhage and sepsis being its most common causes when it occurs. A 27-year-old multiparous woman presented to our Emergency Department at 37 weeks of gestation at the end of the second stage of labor. Vaginal delivery was completed without complications, however, immediately postpartum, inadequate uterine contraction led to hemodynamic instability. Conservative measures failed to control the hemorrhage, leading to the decision to perform an obstetric hysterectomy to restore stability. The surgical procedure was successful, however, progressive renal dysfunction with rising creatinine levels occurred postoperatively, prompting the initiation of hemodialysis sessions, which continued for six days until the full restoration of kidney function. This report describes the occurrence of AKI as a result of postpartum hemorrhage following vaginal delivery and outlines its key risk factors, in addition to diagnosis and treatment options. Proper understanding of these factors can significantly contribute to ensuring maternal health and optimizing perinatal outcomes.

## Introduction

Postpartum hemorrhage (PPH) is defined as blood loss exceeding 500 mL within 24 hours after vaginal delivery, greater than 1000 mL following cesarean section, or any blood loss that may lead to hemodynamic instability in the postpartum patient [1]. PPH is a relatively common obstetric complication and remains the leading cause of maternal morbidity and mortality worldwide, accounting for approximately 27% to 60% of all maternal deaths [2]. Sepsis and hemorrhage, whether occurring during labor, immediately postpartum, or throughout pregnancy, are the most frequent causes of pregnancy-related acute kidney injury (AKI) [3].

AKI following childbirth is a heterogeneous condition, primarily characterized by a sudden and rapid decline in the glomerular filtration rate (GFR) of the postpartum patient, occurring shortly after delivery [4]. Pregnancy-related AKI is uncommon; however, its reported incidence among pregnant women has risen from 0.04% in 2006 to 0.12% in 2015 [5]. This increase is believed to be associated with higher pregnancy rates among older women in developed countries and inadequate prenatal care in developing countries [6].

This case report describes the development of AKI as a result of massive PPH following vaginal delivery, necessitating an emergency obstetric hysterectomy. Additionally, it highlights its key risk factors and explores the modern approaches to its diagnosis and treatment.

## Case presentation

A 27-year-old multiparous woman with a history of two previous cesarean sections presented at the Emergency Department of the General Hospital of Trikala, Trikala, Greece, at 37 weeks of gestation. She reported severe abdominal pain accompanied by mild vaginal bleeding. The patient had not received regular prenatal care at our hospital or any other maternity center, had no laboratory test records, and had not undergone prenatal screening. The only obstetric ultrasound she provided had been performed at 29 weeks of gestation and showed a posteriorly positioned marginal placenta. Her body weight was within the normal range (BMI = 22.5). She was a non-smoker. Her medical history revealed no episodes of bleeding during pregnancy. She reported no hypertension, hyperlipidemia, or diabetes, nor did she experience preeclampsia in her previous pregnancies.

A vaginal examination confirmed that she was in active labor, with almost complete cervical dilation (9 cm) and intact fetal membranes. She was afebrile, with normal blood pressure (110/70 mmHg) and heart rate (95 bpm). Laboratory test results from the emergency assessment upon admission were within normal limits (Table 1).

**Table 1:** The results of our patient's laboratory tests The sudden increase in serum creatinine, combined with the sharp drop in hemoglobin, established the diagnosis of acute kidney injury of hemorrhagic etiology. OH: Obstetric hysterectomy; Ht: Hematocrit; Hb: Hemoglobin; PLT: Platelets; WBC: White blood cells; NEUT: Neutrophils; APTT: Activated partial thromboplastin time; INR: International normalized ratio; FIB: Fibrinogen; Glu: Glucose; Cr: Creatinine; Na+: Sodium; K+: Potassium; TBIL: Total bilirubin; SGOT: Serum glutamic oxaloacetic transaminase; SGPT: Serum glutamate pyruvate transaminase

Laboratory tests	Entrance to the maternity ward	Entrance to the operating room	Six hours after OH	First postoperative day after OH	Second postoperative day after OH	Fourth postoperative day after OH	Normal laboratory values
Ht	41.2%	27.3%	23.8%	24.3%	27.8%	26.4%	37.7-49.7%
Hb	13.6 gr/dL	8.9 gr/dL	7.6 gr/dL	8.4 gr/dL	9.1 gr/dL	8.9 gr/dL	11.8-17.8 gr/dL
PLT	263x10^3^/mL	187x10^3^/mL	149x10^3^/mL	141x10^3^/mL	152x10^3^/mL	191x10^3^/mL	150-350 x10^3^/mL
WBC	7.37x10^3^/mL	12.71x10^3^/mL	21.31x10^3^/mL	18.67x10^3^/mL	13.51x10^3^/mL	8.24x10^3^/mL	4-10.8 x10^3^/mL
NEUT	69.7%	83.1%	94.1%	85.4%	80.1%	69.1%	40-75%
APPT	27.4	33.4	36.9	34.6	31.9	29.4	24.0-35.0 sec
INR	0.97	1.15	1.31	1.24	1.11	1.01	0.8-1.2
FIB	287 mg/dL	236 mg/dL	185 mg/dL	171 mg/dL	187 mg/dL	195 mg/dL	200-400 mg/dL
Glu	124 mg/dL	121 mg/dL	87 mg/dL	85 mg/dL	73 mg/dL	79 mg/dL	75-115 mg/dL
Cr	0.47 mg/dL	0.47 mg/dL	1.55 mg/dL	2.11 mg/dL	2.07 mg/dL	1.85 mg/dL	0.40-1.10 mg/dL
Na^+^	-	135.9 mmol/L	132.1 mmol/L	137.2 mmol/L	139.6 mmol/L	-	136-145 mmol/L
K^+^	-	4.41 mmol/L	3.71 mmol/L	3.95 mmol/L	3.97 mmol/L	-	3.5-5.1mmol/L
TBIL	-	0.83 mg/dL	1.27 mg/dL	1.21 mg/dL	0.96 mg/dL	-	0-1.2 mg/dL
SGOT	-	25 IU/L	26 IU/L	29 IU/L	27 IU/L	-	5-33 IU/L
SGPT	-	12 IU/L	14 IU/L	15 IU/L	14 IU/L	-	10-37 IU/L

After artificial rupture of the membranes and the intravenous (IV) administration of a minimal dose of oxytocin (10 units of oxytocin in 1000 mL of Ringer’s solution, infused at a rate of 30 mL/h), vaginal delivery was completed uneventfully within 20 minutes without episiotomy. The newborn weighed 2560 grams. Following placental expulsion, no vaginal or cervical tears were observed.

**Figure 1:**
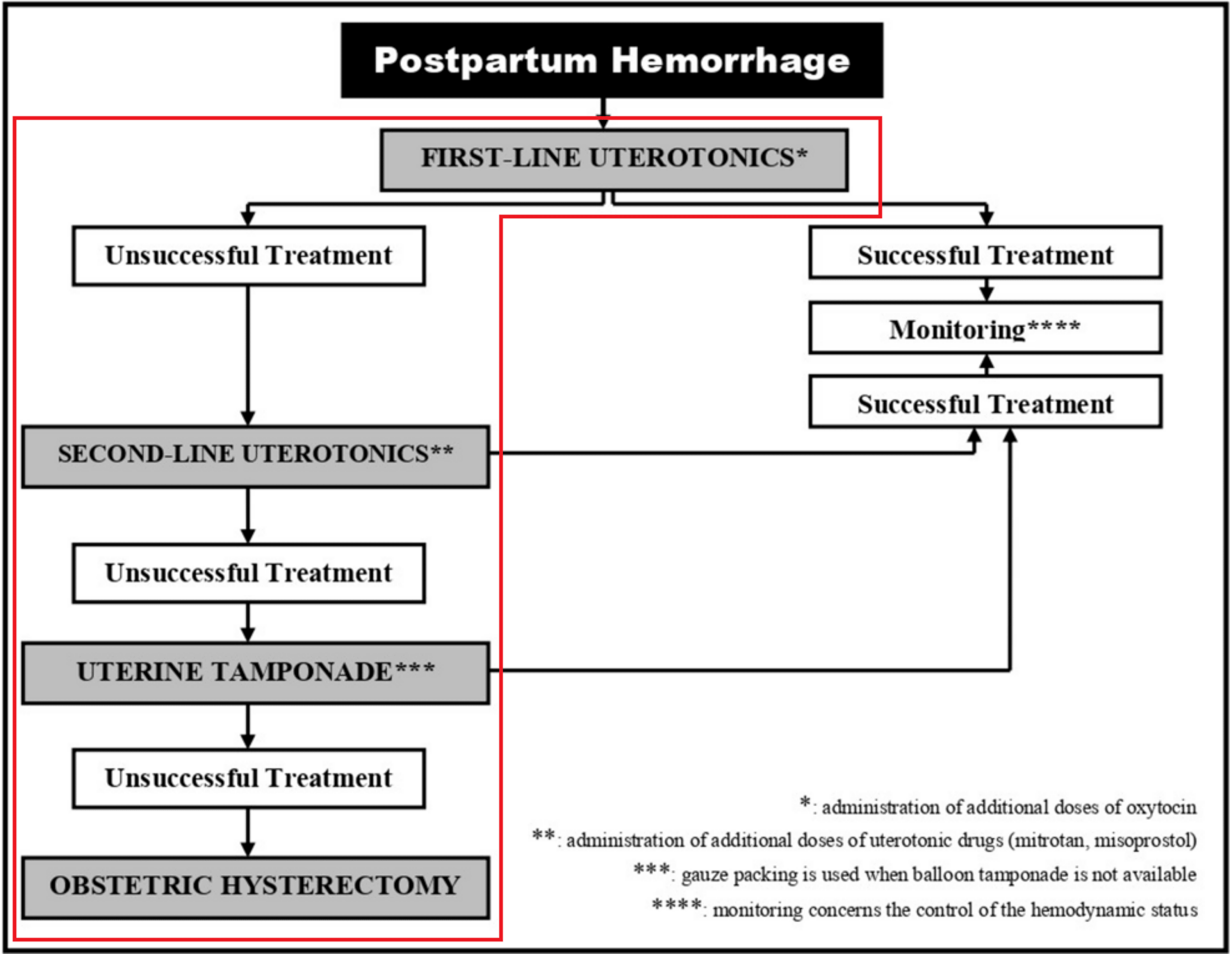
Flowchart of the management protocol for postpartum hemorrhage in our Clinic Red outline: steps followed during the management of the present case.

During postpartum monitoring, increased lochia were noted, along with reduced uterine tone and a fundal height palpated above the umbilicus. Immediate vaginal examination revealed the presence of large blood clots. Additional doses of first-line uterotonic agents (oxytocin) were administered as a 10-unit IV bolus, followed by a continuous infusion at an increased rate of 150 mL/h. However, this intervention was unsuccessful, prompting the use of second-line medication, namely IV methylergonovine (Mitrotan) at a dose of 0.2 mg bolus and oral misoprostol (Cytotec) at a dose of 400 mcg, which also failed to control the hemorrhage. The patient developed hemodynamic instability (blood pressure 70/50 mmHg, heart rate 115 bpm), prompting her transfer to the operating room. Under general anesthesia, a digital uterine examination ruled out traumatic uterine wall injuries. Uterine-cervical-vaginal tamponade using gauze packing failed to achieve hemostasis. A uterine balloon tamponade was not available at our hospital. The persistent hemodynamic instability and the failure of conservative measures necessitated an emergency obstetric hysterectomy. The management protocol for PPH applied in our Clinic, in addition to the route followed in this particular case, is depicted in Figure 1.

Upon induction of anesthesia, the patient received a transfusion of two units of blood and one unit of plasma. The surgical procedure was performed via laparotomy and proceeded smoothly and uneventfully, without notable intraoperative findings. The total estimated blood loss since delivery was approximately 1800 mL. Postoperatively, an additional transfusion of three units of blood and two units of plasma were performed, with five units of blood, three units of fresh-frozen plasma and approximately 3000 mL in fluids (Ringer’s solution) having been administered cumulatively up to that point. Urinary output monitoring revealed oliguria (320 mL/24 hours). Renal, ureteral, and bladder ultrasound findings were unremarkable. Serum creatinine levels were elevated during the first two postoperative days (Table 1). The nephrology team diagnosed AKI and recommended renal replacement therapy with hemodialysis. The patient and her newborn, both in stable condition, were discharged on the sixth postoperative day. Hemodialysis was continued by the nephrology team until full recovery of renal function.

## Discussion

The present report describes a case of AKI as a result of hemodynamic instability following uncontrolled PPH and surgery. While the condition was successfully resolved with hemodialysis in the present case, AKI constitutes a dangerous clinical entity with potentially long-term, irreversible repercussions, especially in the context of pregnancy and the postpartum period.

Pregnancy-related AKI may be induced by a plethora of potential risk factors, including those not unique to pregnancy, such as vascular disease caused by advanced age, smoking, obesity, hyperlipidemia, hypertension or diabetes. First-trimester pregnancy-specific factors include dehydration and malaise due to hyperemesis gravidarum and septic abortion leading to glomerular necrosis, the latter being more common in developing countries [7]. Second and third-trimester pregnancy-related risk factors include preeclampsia, HELLP (Hemolysis, Elevated Liver Enzymes, and Low Platelet count) syndrome, acute fatty liver of pregnancy, thrombotic microangiopathies (e.g., thrombotic thrombocytopenic purpura), atypical hemolytic-uremic syndrome, urinary tract infections, pyelonephritis and placental abruption [6]. Our patient had none of the aforementioned risk factors, being young, relatively fit, a non-smoker and having no history of preeclampsia, hyperlipidemia, or diabetes, with the underlying cause in this case being the massive PPH, which necessitated an emergency obstetric hysterectomy. The study by Mir et al. studied the etiology of AKI in the postpartum period and, after confirming the rarity of the condition (approximate incidence 2%), ultimately identified PPH as the second most common etiology, after sepsis [8], similar to our own case, thus verifying our observation.

Diagnosis of AKI is based on elevated serum creatinine levels, which may be challenging during pregnancy, as an increase may be masked by the naturally lower baseline creatinine levels due to increased glomerular filtration occurring in pregnancy [9]. The absence of recorded pre-pregnancy creatinine measurements further complicates early and accurate diagnosis [10]. A sudden increase in serum creatinine >0.3 mg/dL from baseline or >1.5 times the reference value or reduced urine output (<0.5 mL/kg body weight per hour for 6-12 hours) or the need for hemodialysis are currently criteria for the diagnosis of AKI in pregnant women [11,12]. Subsequent investigations include a renal ultrasound to rule out obstructive AKI and biopsy in case of inconclusive laboratory and image findings [11,13]. In our case, the sudden rise in serum creatinine (1.55 mg/dL) and oliguria (320 mL/24 hours) clearly indicated the presence of AKI, later confirmed by the need for hemodialysis. The same criteria were utilized by Mir et al., with oliguria being the most common symptom, present in 100% of cases and thus constituting a reliable clinical marker [8]. Furthermore, in the study by Coles et al., the authors also highlighted the utility of specific risk factors as a means of early diagnosis and prediction of the occurrence of AKI in the post-partum period [14]. Amongst others, they demonstrated that patients with pre-eclampsia, emergency cesarean section and severe PPH had 14, 9 and 6 times higher risk of AKI compared to controls [14]. The latter is in agreement with our observations, as the patient did have severe, uncontrollable PPH prior to developing AKI, indicating that PPH could be used as an early predictor.

Management of AKI in pregnancy is challenging and requires a multidisciplinary approach, via collaboration of nephrologists, obstetricians, neonatologists and intensivists in order to ensure successful outcomes [15]. Treatment success is based on accurate determination of the underlying cause [16], with an estimated that two-thirds of pregnant patients with AKI recovering fully within 12 weeks postpartum with etiology-based treatment [17]. Treatment options include antibiotics in cases of septic abortion [18], IV fluid administration for pre-renal AKI (with careful monitoring in preeclampsia or severe hemorrhage in order to avoid pulmonary edema) and potentially early delivery in cases of severe preeclampsia, HELLP syndrome, or acute fatty liver of pregnancy. In severe AKI with multi-organ complications, renal replacement therapy, including peritoneal dialysis or hemodialysis, may be considered [16], as it has been shown to significantly improve prognosis for both mother and the neonate [19]. In our patient, IV fluids, fresh-frozen plasma, and concentrated red blood cells were unable to reverse the hemodynamic instability induced by PPH, necessitating the more drastic measures of emergency obstetric hysterectomy and hemodialysis. The prognosis of AKI, while poor, with maternal mortality declined from 72% in the period 1984-1994 to 19% between 1995 and 2005, in large part thanks to improvements in efficacy and availability of treatment and critical care [20,21]. Even if AKI is successfully treated in the acute phase, there is a risk of progression to end-stage renal failure in the mother [22]. This was the case in the study by Mir et al., where 14% of women with postpartum AKI ended up requiring hemodialysis on a permanent basis, with renal biopsies indicating cortical necrosis [8]. However, they did stress that the majority of patients managed to fully recover, even in the presence of advanced renal injury in the beginning [8], confirming our observations with the present case and highlighting the value of immediate treatment in preserving renal functionality after the acute phase and improving long-term outcomes.

## Conclusions

Pregnancy-related AKI is a rare obstetric complication. The absence of vascular disease risk factors can make early diagnosis of non-hemorrhagic pregnancy-related AKI more challenging for clinicians. Severe PPH is a major cause of severe AKI in pregnant women. In our patient, AKI was the result of massive bleeding after vaginal delivery, for the treatment of which it was deemed necessary to perform an obstetric hysterectomy followed by renal replacement therapy. Timely diagnosis and effective management of obstetric hemorrhage-associated AKI, including hemodialysis, are crucial in reducing maternal and perinatal morbidity and mortality.
